# Treatment of Behçet's Disease: An Algorithmic Multidisciplinary Approach

**DOI:** 10.3389/fmed.2021.624795

**Published:** 2021-04-28

**Authors:** Erkan Alpsoy, Pietro Leccese, Giacomo Emmi, Shigeaki Ohno

**Affiliations:** ^1^Department of Dermatology and Venereology, School of Medicine, Akdeniz University, Antalya, Turkey; ^2^Rheumatology Department of Lucania, Rheumatology Institute of Lucania, San Carlo Hospital of Potenza and Madonna delle Grazie Hospital of Matera, Potenza and Matera, Italy; ^3^Department of Experimental and Clinical Medicine, University of Firenze, Firenze, Italy; ^4^Ophthalmology Center, Aishin Memorial Hospital, Sapporo, Japan; ^5^Department of Ophthalmology, Faculty of Medicine, Graduate School of Medicine, Hokkaido University, Sapporo, Japan

**Keywords:** algorithms, therapeutics, morbidity, mortality, Behçet's disease

## Abstract

Behçet's disease (BD) is a chronic, relapsing inflammatory, multisystem disease of unknown etiology. The disease has a wide clinical spectrum of mucocutaneous lesions and ocular, vascular, articular, neurologic, gastrointestinal and cardiac involvement. Although the number of effective drugs used in the disease's treatment has increased in recent years, BD is still associated with severe morbidity because of mainly mucocutaneous, articular and ocular symptoms and an increased mortality because of large vessel, neurological, gastrointestinal and cardiac involvement. Many factors are associated with a more serious course, such as male gender and a younger age of onset. While the severity of the disease is more pronounced in the first years of the disease, it decreases in most patients after the age of forties. The primary goal of treatment should be the prevention of irreversible organ damage. Therefore, early diagnosis and appropriate treatment and close follow-up are mandatory to reduce the morbidity and mortality of the disease. Treatment varies depending on the organ involved and the severity of the involvement. For all these reasons, the treatment should be personalized and arranged with a multidisciplinary approach according to the organs involved. Treatment is mainly based on suppression of the inflammatory attacks of the disease using local and systemic immunomodulatory and immunosuppressive drugs. In this review, based on the mainly controlled studies and personal experience in clinical practice and basic research in this field, we propose a stepwise, symptom-based, algorithmic approach for the management of BD with a holistic perspective.

## Introduction

Behçet's disease (BD) is a chronic, relapsing and debilitating inflammatory multisystem disease of unknown etiology (1). Although the disease has been defined as a trisymptom complex characterized by recurrent oral ulcers (OU), genital ulcers (GU), and uveitis, subsequent studies have shown that BD spectrum includes different clinical phenotypes affecting the joints, central nervous system, major blood vessels, heart, and gastrointestinal tract (2). Although BD is more common in “Silk Road” populations, it has a universal distribution (3). The interplay between a complex genetic background and both innate and adaptive immune system is related to the BD clinical features (4–6). Due to the lack of a universally recognized pathognomonic laboratory test, the diagnosis is based on clinical criteria. The International Study Group criteria are the most widely used and well-accepted criteria among the experts of this field (7). Recently, a new set of criteria including vascular and neurological involvement has also been proposed through an international collaborative effort (8). Given the complexity of the disease therapeutic approach varies according to the different clinical involvement and phenotypes.

### Clinical Features

#### Mucocutaneous Lesions

Mucocutaneous lesions are the distinctive clinical feature of BD. Their frequent occurrence at the beginning or at any stage of the disease emphasizes the importance of mucocutaneous lesions for diagnosis. OU, GU and cutaneous lesions, together with ocular and articular involvement, are the most frequent clinical manifestations (3). Mucocutaneous lesions can cause serious problems in patients' quality of life and psychosocial worlds. OU, GU, erythema nodosum (EN)-like lesions, papulopustular lesions (PPL), or other less common cutaneous lesions (e.g., extragenital ulcers, Sweet's syndrome-like and pyoderma gangrenosum-like lesions) may cause significant pain and/or loss in function (3, 9–11).

#### Articular Involvement

Articular involvement is observed in approximately half of the patients and is characterized by non-deforming arthritis, which often presents with monoarticular or oligoarticular pattern. It is usually transient, with episodes lasting from a few days to weeks. The knee is the most frequently affected joint, followed by the ankle, wrist and elbow (12). Diri et al. (13) reported that papulopustular lesions (PPL) are seen more frequently in BD patients with arthritis.

#### Ocular Involvement

Ocular involvement, one of the most serious and disabling complications of BD, is seen in approximately half of the patients. It is characterized by recurrent, explosive inflammatory attacks that can lead to blindness if left untreated. Recently, visual prognosis has improved significantly with the use of new treatments (e.g., anti TNF-alpha agents) (14). Ocular involvement is more common and severe in male patients (15). Bilateral involvement is seen in 86% of patients (15). Ocular lesions comprise anterior uveitis, intermediate uveitis, and more frequently posterior uveitis and panuveitis. Repeated intraocular inflammation causes major ocular complications (e.g., secondary cataract, secondary glaucoma, cystoid macular edema) often causing severe decreased vision or blindness (16). Therefore, the strategy for treating ocular BD should be not only for the suppression and treatment of uveitis but also for the prevention of ocular complications (16, 17).

#### Vascular Involvement

Vascular involvement is one of the most important causes of mortality in BD. Although BD can affect vessels of any size and type (18), venous system is the major affected site, and superficial and deep vein thrombosis are the most frequent type of vascular involvements. Thromboses of the inferior and superior vena cava, dural sinuses and Budd-Chiari syndrome can also be seen and are associated with poor prognosis. Although rare, pulmonary artery aneurysm is the most common cause of death (19).

#### Neurological Involvement

Neurological involvement is one of the most serious complications of the disease because of its severe prognosis. Neurological symptoms affecting 5–10% of all patients are more common in men. It is distinguished in the parenchymal (pNBD) and non-parenchymal form. NBD can be characterized by single-acute attack, relapsing-remitting or chronic progressive course. Rapid disease progression, history of frequent relapses and presence of cerebrospinal fluid pleocytosis are associated with poor prognosis (20). The therapeutic approach depends on the type of involvement and should be started immediately.

#### Gastrointestinal Involvement

Gastrointestinal involvement is reported in ~3–16% of patients and is more common in far eastern countries. It is characterized by punched-out mucosal ulcers occurring predominantly ileocecal region, although it can occur throughout the gastrointestinal tract. Abdominal pain, nausea, vomiting, diarrhea and bleeding are the most common symptoms. Deep punched-out ulcers are responsible for the most common intestinal complications such as severe bleeding and perforation. Intestinal lesions are considered as being a poor prognostic factor (21).

### Course

BD follows a chronic course with unpredictable inflammatory attacks and remission periods. Male gender and early age of onset are associated with severe disease. Each or any combination of the mucocutaneous, articular, and ocular symptoms can cause significant physical and psychological morbidity. BD has an increased mortality rate, especially in young men, due to involvement of the pulmonary artery and other large vessels, neurological, gastrointestinal and cardiac involvement (22). BD usually starts with relatively mild symptoms; severe involvement occurs later (11, 23).

### Treatment

Treatment varies depending on involved organ/s, the severity and duration of involvement, the frequency of attacks, gender and patient's age. At present, no specific recommendations based on gender or age exist for all the manifestations of BD; however, age and early disease can influence the treatment of ocular involvement. Nevertheless, the primary goal of treatment should be rapidly to suppress and prevent new inflammatory attacks to avoid irreversible organ damage, especially in the early, active stages of BD. Randomized controlled trials are limited to mucocutaneous, articular and ocular involvement. In this review, we propose a symptom-based, algorithmic treatment approach. Although the recommendations are mainly based on controlled studies, important studies, guidelines, expert reviews and finally, our personal experience in clinical practice is also included.

### Treatment Algorithms

Activity spectrum of topical and systemic therapeutic agents (24–59) on BD in randomized, controlled studies is summarized in Table 1.

**Table 1:** The main topical and systemic therapeutic agents used in the treatment of Behçet's disease in randomized, controlled studies.

**Topical treatments**	**Dose ∥ duration ∥ patient number**	**Result and reference**
Cyclosporine vs. Placebo	70 mg per g of orobase ∥ 8 w ∥ 24	No significant difference on OU between the treatment arms (24)
Interferon alpha vs. Placebo	1 × 10^5^ U/g thrice a day ∥ 24 w ∥ 30	Not effective on OU (25)
Interferon alpha vs. Placebo	1,000/2,000 IU a day ∥ 12 w ∥ 84	No beneficial effects on reducing the total OU burden (26)
Pentoxifylline + Colchicine vs. Colchicine	1,000 mg/d (4 divided doses) ∥ 14 d ∥ 21	Significant decrease in the duration and pain of OU in pentoxifylline group (27)
Pimecrolimus + Colchicine vs. Colchicine	Twice a day + 1–2 g/d ∥ 4 w ∥ 38	Significant decrease in the pain severity of GU in pimecrolimus group (28)
Pimecrolimus vs. Placebo	Twice a day ∥ 4 w ∥ 45	Accelerates the healing process of GU (29)
Sucralfate vs. Placebo	4 times a day ∥ 3 mo ∥ 40	Decreases the frequency, healing time and pain of OU, and the healing time and pain of GU (30)
Triamcinolone acetonide vs. Phenytoin	Thrice a day ∥ 1 w ∥ 60	Shown to be more effective than phenytoin on OU (31)
**Systemic treatments**	**Dose** **∥** **duration** **∥** **patient number**	**Result and reference**
Acyclovir vs. Placebo	800 mg/d ∥ 12 w ∥ 44	Not effective on the frequency and severity of OU and GU or other disease features (32)
Apremilast vs. Placebo	30 mg/ twice a day ∥ 12 w ∥ 111	Reduces the numbers of OU and GU, and pain of OU (33)
Azathioprine vs. Placebo	2.5 mg/kg/d ∥ 2 y ∥ 63	Reduces the occurrence of OU, GU, arthritis and ocular symptoms. Prevents the development of new eye disease (34)
Azapropazone	900 mg/d ∥ 3 w ∥ 57	Not effective in controlling the arthritis (35)
Cyclophosphamide + Corticosteroids vs. Corticosteroids	1 g/m^2^ /mo + 0.5 mg/kg ∥ 6 mo ∥ 35	Combined treatment of Cyclophosphamide and corticosteroids more effective in eye disease than corticosteroids alone (36)
Colchicine vs. Placebo	1 mg/d ∥ 9 mo ∥ 28	Decreases the frequency of EN, and effective on arthralgia (37)
	1–2 mg/d ∥ 2 y ∥ 116	Reduces the occurrence of GU, EN and arthritis in women, and the occurrence of arthritis in men (38)
	1 mg/d ∥ 4 mo ∥ 169	Significant improvement in disease activity index, and OU, GU, EN and PPL in both gender (39)
Colchicine vs. Colchicine + Benzathine penicillin	1–2 mg/d + 1.2 MU /3 w ∥ 2 y ∥ 154	Combined treatment more effective in reducing frequency of arthritic episodes, duration of OU and EN and the frequency of GU (40)
Corticosteroids vs. Placebo	40 mg/every 3 w ∥ 27 w ∥ 41	Decrease the frequency of EN in women (41)
Cyclosporine vs. Colchicine	10 mg/kg/d + 1 mg/d ∥ 4 mo ∥ 96	Cyclosporine more effective on the severity and frequency of OU, GU and PPL. Superior to colchicine in decreasing the frequency and severity of ocular attacks (42)
Cyclosporine vs. conventional treatments (prednisolon, chloroambucil)	5–10 mg/kg/d ∥ 3 y ∥ 40	Cyclosporine more effective than conventional therapy in ocular disease, however, conventional therapy superior to Cyclosporine in controlling OU, GU and arthritis (43)
	0 mg/kg/d ∥ 1 y ∥ 35	Improvement of hearing loss in 25% of patients receiving Cyclosporine treatment (44)
Cyclosporine vs. conventional treatments (prednisolon, Azathioprine)	5 mg/kg/d ∥ 6 mo ∥ 76	Cyclosporine more effective than conventional therapy in OU, GU, cutaneous lesions, STP as well as articular and neurologic symptoms (45)
Cyclosporine vs. Cyclophosphamide	5 mg/kg/d ∥ 1 y ∥ 23	A significant improvement in VA during the first 6 mo in Cyclosporine group compared with Cyclophosphamide (46)
Daclizumab vs. Placebo	1 mg/kg /2w for 6 weeks ∥ 6 mo ∥ 17	No beneficial effect in comparison with placebo (47)
Dapson vs. Placebo	100 mg/d ∥ 12 w ∥ 20	Effective on the number, healing time and frequency OU, number of GU, and frequency of EN and PPL. Supresses arthritis and epididymitis (48)
Etanercept vs. Placebo	25 mg/d-2 x/w ∥ 4 w ∥ 40	Reduces the occurrence of OU, nodular skin lesions and PPL (49)
Interferon-α2a vs. placebo	6 MU/d-3 x/w ∥ 12 w ∥ 50	Effective on pain and healing time of OU, and frequency of GU and PPL (50)
Interferon-α2b (pegilated) vs. glucocorticoids and immunosuppressives	0.3 μg/kg/w ∥ 26 w ∥ 72	Significant reduction in corticosteroid dose at 1 year with the addition of peginterferon-α-2b to the drug regime in patients with BD with ocular and systemic involvement (51)
Interferon-α2a vs. Cyclosporine	3–9 MU/d-3 x/w vs. 3–5 mg/kg ∥ 1 y ∥ 26	More patients were in remission in the IFN alpha arm. The switches from Cyclosporine to IFN alpha was significantly greater (52)
Isotretinoin vs. Placebo	20 mg/d ∥ 12 w ∥ 30	Significant improvement in the clinical manifestations index, and OU and skin manifestations parameters (53)
Levamisole vs. Placebo	3 × 50 mg, 2 days/w ∥ 8 w ∥ 47	Improvement in OU and GU together with arthritis and uveitis (54)
Rebamipide vs. Placebo	300 mg/d ∥ 6 mo ∥ 35	Reduces the number of OU and pain (55)
Rituximab vs. Cytotoxic combination therapy	2 × 1,000-mg courses (15-day interval) ∥ 6 mo ∥ 20	A significant improvement in total adjusted disease activity index in rituximab group (56)
Secukinumab vs. Placebo	300 mg/2w or 300 mg/mo ∥ early termination ∥ 118	No statistically significant differences in uveitis recurrence; beneficial effect in reducing the use of concomitant immunosuppressive medication (57)
Thalidomide vs. Placebo	100–300 mg/d ∥ 6 mo ∥ 96	Sustained remission of OU and GU and PPL (58)
Zinc sulfate vs. Placebo	300 mg/d ∥ 6 mo ∥ 30	Significant improvement in the clinical manifestations index of mucocutaneous lesions (59)

*EN, erythema nodosum-like lesions; GU, genital ulcers; OU, Oral ulcers; PPL, papulopustular lesions; STP, superficial thrombophlebitis*.

#### Topical Treatment

Corticosteroids alone (triamcinolone acetonide in oral paste or dexamethasone ointment) for OU and in combination with antiseptics (e.g., fusidic acid/betamethasone) for GU are useful, especially when used in the early stages of these lesions (31, 60, 61). Sucralfate reduces pain while accelerating healing in both OU and GU. Pentoxifylline 5% gel decreases the duration and pain of OU (27). Pimecrolimus is an effective and safe compound in GU treatment (28, 29, 61). Wet dressings such as aluminum acetate 3–5% are useful in the early stages of EN-like lesions and STP (60).

Since OU because of BD is similar to recurrent aphthous stomatitis (RAS) the treatments recommended for RAS can be applied to OU of BD. Topical antibiotics (e.g., tetracyclines and their derivatives), antimicrobial agents (chlorhexidine), amlexanox, triclosan are beneficial by accelerating healing and can be used first-line treatments. Hydroxypropyl cellulose, diclofenac, lidocaine, silver nitrate, CO_2_ laser, Nd:YAG laser is useful in decreasing pain and can be used as second-line options (3, 60).

#### Systemic Treatment

##### 1-Mucocutaneous Manifestations

**Figure 1:**
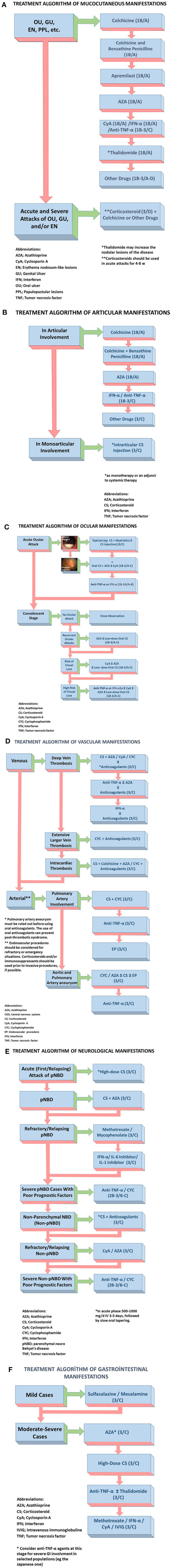
Treatment algorithms of mucocutaneous **(A)**, articular **(B)**, ocular **(C)**, vascular **(D)**, neurologic **(E)** and gastrointestinal **(F)** symptoms. In the flow charts all treatments were placed one under the other in the right columns. Algorithms start from the boxes in the upper left corner. The green arrow means “yes,” the red arrow means “no”.

Colchicine can be used as the first-line treatment of mucocutaneous lesions (22, 37–39, 62). Benzathine penicillin can be added to colchicine to increase the effectiveness (40). Apremilast is another important alternative with proven efficacy in the treatment of mucocutaneous lesions (33, 63). Azathioprine can be used in patients inadequately controlled with the treatments above (34). Cyclosporine, interferon (IFN)-α and anti-tumor necrosis factor (TNF)-α agents are effective in patients who cannot be controlled with previous treatments (42, 45, 49, 50, 64). Thalidomide is often helpful (58). However, it should be used with caution in selected patients because of potential side effects. Levamisole, dapsone, rebamipide, zinc sulfate, isotretinoin, methotrexate, pentoxifylline, secukinumab and ustekinumab are other alternatives (22, 48, 53–55, 59, 62, 64–66) (Figure 1A).

Levamisole (54), dapsone (48), zinc sulfate (59), and isotretinoin (53) treatments could be considered in the earlier steps of the algorithm because of the availability of controlled studies. However, there have not been publications about these treatments in the recent years. Also, newer and more effective treatments have appeared in recent years. Since the effect of rebamide is limited to OU, it could not be evaluated at earlier steps in the algorithm (59).

In acute and severe attacks of mucocutaneous lesions (e.g., major OU, GU, and/or EN-like lesions), corticosteroids (prednisolone, initial dose 40–60 mg daily for 2–4 weeks, tapered over the ensuing 4–6 weeks) can be used as an effective treatment. In this case, corticosteroids are used in addition to previous treatment. If the patient does not receive any systemic treatment, it would be more appropriate to combine it with a treatment such as colchicine (3, 62).

##### Articular Involvement

Colchicine is often chosen as the first-line treatment to prevent arthritis attacks (38). In patients unresponsive to colchicine monotherapy, the addition of benzathine penicillin may be beneficial (67). Azathioprine can be considered in patients with recurrent arthritis and/or with resistant disease. IFN-α and anti-TNF-α agents may be used in even more severe but uncommon cases (68–72). Although systemic corticosteroids and non-steroidal anti-inflammatory drugs are widely used to treat arthritis-related symptoms, the evidence from controlled studies with azapropazone or intramuscular methylprednisolone acetate was disappointing (35, 41). Intraarticular corticosteroid injections can be considered in patients with monoarthritis even as an adjunct to systemic therapy, but evidence from randomized clinical trials is lacking (22). Limited data suggests ustekinumab, secukinumab and anti-IL-1 agents as alternative treatment options (65, 73, 74) (Figure 1B).

##### Ocular Involvement

Treatment of ocular BD firstly needs to suppress and manage acute inflammation in the anterior uvea, retina, retinal vessels, choroid and optic disc in the exacerbation stage. Since ocular lesions suddenly reappear with unclear triggering factors, it is important to prevent subsequent ocular inflammatory attacks during the ocular convalescent stage (16, 17) (Figure 1C).

In acute ocular attack, topical eye drops of corticosteroid and mydriatics should be given. Subconjunctival corticosteroid injection may sometimes be required in patients with an acute attack limited to the anterior part of the eye. Depending on the severity of ocular fundus inflammation, corticosteroid injection of the posterior sub-Tenon and oral corticosteroid therapy can be recommended besides topical corticosteroids and mydriatics. In unresponsive cases or in the presence of posterior segment involvement, azathioprine and/or Cyclosporine should be initiated in addition to systemic corticosteroids (22). Anti-TNF-α agents or IFN-α should be the treatments to be considered in the next step in patients who cannot be controlled by this treatment or in those with acute sight-threatening ocular presentation (16, 17, 22, 62).

In the convalescent stage, if there is no ocular inflammatory attack, treatment is unnecessary and only close inspection of clinical signs is sufficient. Azathioprine with or without low-dose corticosteroids should be given in those who have recurrent ocular attacks. If clinical convalescence is kept with no ocular recurrence for 6 months or longer, azathioprine can be continued. However, if the patient has recurrent ocular attacks, especially in the ocular fundus, there may be a risk of decreased visual function. In this case, cyclosporine should be started with or without previous therapy (17). Anti-TNF-α agents (infliximab or adalimumab) or IFN-α should be given with or without oral cyclosporine, azathioprine and/or oral prednisolone if previous treatments are insufficient (75–77). It is difficult to avoid decreased visual function in patients inadequately controlled with anti-TNF-α agents or IFN-α; here, other new treatments such as IL-1 inhibitors should be considered (78).

##### Vascular Involvement

The treatment of vascular BD manifestations differs according to the involved district, and to the specific type of event. However, in BD patients, different types of vascular involvement can coexist in the same patients, not necessarily simultaneously. This peculiar aspect suggests that all the vascular events have similar pathogenic pathways, mainly driven by inflammatory mechanisms (1, 79–81). The inflammatory nature of vascular events in BD, deeply influences the treatment approach (Figure 1D).

##### Venous Involvement

In patients with venous involvements of typical sites (deep vein thrombosis of the legs and arms), corticosteroids and immunosuppressive agents represent the mainstay treatment (79–82). Immunosuppressive therapy is pivotal to prevent recurrences and to reduce the risk of post-thrombotic syndrome, whereas the use of anticoagulants in deep vein thrombosis is still controversial (19). According to current recommendations, there is no valid data to prefer one immunosuppressant over another. However, evidence suggests the choice of azathioprine, Cyclosporine or cyclophosphamide (22).

In patients with refractory venous thrombosis, anti-TNF-alpha agents, alone or in combination with traditional DMARDs (83, 84), or interferon-alpha, can be considered (85), eventually in association with anticoagulants. In the latter case, patient's specific bleeding risk should be considered, and the presence of aneurysms should be always evaluated.

Conversely, the association of anticoagulant to immunosuppressive therapy is suggested in case of extensive thrombosis of larger veins, particularly of vena cava; in this case, cyclophosphamide is preferred to the other immunosuppressive agents (22).

Similarly, the triple association of steroids, immunomodulating/immunosuppressive agents (colchicine, eventually combined with azathioprine and/or cyclophosphamide), and anticoagulants seem to be the most effective choice also in case of intracardiac thrombosis (19).

##### Arterial Involvement

Immunosuppressants should always be considered to achieve complete remission and prevent post-operative complications. The concomitant use of anticoagulants might be beneficial, particularly to reduce the risk of post-operative thrombosis (19).

According to EULAR recommendations, patients presenting with pulmonary artery involvement (PAI) should start high-dose corticosteroids and cyclophosphamide, whereas the use of anticoagulants in this condition is negligible. In patients refractory to this first-line treatment, anti-TNF-α agents (mainly infliximab) can represent a life-saving treatment (86). Eventually, embolisation, lobectomy, cavitectomy, and decortication can be considered (87–89).

For aortic and pulmonary artery aneurysm (PAA), pharmacological treatment is mainly based on immunosuppressants, namely cyclophosphamide or azathioprine, mostly in combination with corticosteroids and with surgery (90, 91). Anti-TNF-α agents can be considered for refractory cases (22).

##### Neurological Involvement

In the acute phase of pNBD, the first-line therapy is represented by high-dose intravenous corticosteroids (500–1,000 mg daily for 3–5 consecutive days) followed by slow oral tapering. Decisions about dosage and treatment duration are based on the severity of attack and the clinician's judgment. Therefore, the steroid reduction schedule is not standardized and it should be done accordingly to clinical response (Figure 1E).

To treat pNBD, an immunosuppressive agent such as azathioprine should be started besides high-dose corticosteroids. In clinical practice to assess tolerability to azathioprine it is possible to start with lower doses (1–1.5 mg/kg/day) and increase gradually every 5–7 days up to the maximum therapeutic dosage (2.5 mg/kg/day).

In patients with severe clinical presentation or poor prognostic factors, anti-TNF-α agents or cyclophosphamide can be considered as a first-line therapy. Cyclophosphamide can be administrated orally (1–3 mg/kg/day) or by intravenous pulse (500–1,000 mg/m^2^ every month for 6–9 months). A retrospective study comparing three different therapeutic regimens (corticosteroids alone, azathioprine+corticosteroids, cyclophosphamide + corticosteroids) reported no significant differences in terms of long-term outcome although patients with a severe disability at baseline treated with high-dose corticosteroids plus intravenous cyclophosphamide had a longer event-free survival (92).

Anti-TNF-α agents have been associated with a high response rate. More than 80% of NBD patients showed good clinical response. Therefore, anti-TNF-α agents reduces the risk of relapses and progression of disability (22, 72, 93–95).

Because of limited scientific evidence, other drugs such as IFN-α, methotrexate, mycofenolate mofetile, anti-IL 6 or anti-IL-1 agents should be considered in selected cases as alternative options (73, 96–101).

Cyclosporine seems to be associated with an increased risk of developing pNBD although the reason is unknown. Cyclosporine should be discontinued or avoided in patients with pNBD (64).

The treatment of venous sinus thrombosis is based on high-dose corticosteroids in association with short-term anticoagulation. Usually immunosuppressive treatment is not needed in patients at first episode but should be considered in relapsing cases. Azathioprine, cyclosporine, cyclophamide and anti-TNF-α agents can be used. The choice of treatment should be based on patient's characteristics, disease severity and involvement of other organs. Long-term anticoagulation may be useful in patients with relapsing disease and/or hypercoagulability state (85, 102).

So far, no evidence from controlled studies are available for the treatment of NBD and a phase 3 randomized trial comparing the efficacy and safety of infliximab to that of cyclophosphamide in severe BD is ongoing (ClinicalTrials.gov Identifier: NCT03371095).

##### Gastrointestinal Involvement

The pharmacological treatment of gastrointestinal involvement varies according to its severity. While milder cases should be initially treated with 5-amino salicylate derivatives (e.g., sulfasalazine, mesalamine), azathioprine should be considered in unresponsive or more severe cases (22). Oral or intravenous high-dose corticosteroids should be considered in the most severe cases (21, 103). The true risk-benefit profile of high-dose corticosteroids is still a matter of debate (104) and current evidence on their efficacy in gastrointestinal involvement is inadequate to recommend their routine use in clinical practice (103) (Figure 1F).

In case of severe enteric manifestations poorly controlled by azathioprine, anti-TNF-α agents (infliximab or adalimumab) (105, 106) and/or of thalidomide should be considered (107, 108).

Some evidence suggests that other immunomodulating therapies including methotrexate, interferon, cyclosporine, and intravenous immunoglobulins can also effectively control gastrointestinal symptoms (103). However, given the poor evidence supporting their routine use, these treatments should be considered as a fourth-line option for gastrointestinal involvement.

## Conclusions

Because of the high incidence of vital organ involvement, regular follow-up and appropriate management of BD is mandatory. Because of its multisystemic nature, collaboration among the related specialities to improve patient outcomes is requisite. In this respect, there is a need for organizations where physicians experienced in the disease can serve together. Multi-center, large-series controlled studies should be encouraged to get optimal patient management, especially in organ involvement with high mortality (e.g., vascular, neurological, gastrointestinal).

## Author Contributions

All authors: writing, revision of the manuscript, acquisition of clinical data, and conception and design.

## Conflict of Interest

The authors declare that the research was conducted in the absence of any commercial or financial relationships that could be construed as a potential conflict of interest.
